# Genetic Diversity and Association Mapping of Grain-Size Traits in Rice Landraces from the Honghe Hani Rice Terraces System in Yunnan Province

**DOI:** 10.3390/plants12081678

**Published:** 2023-04-17

**Authors:** Mengli Ma, En Lei, Tiantao Wang, Hengling Meng, Wei Zhang, Bingyue Lu

**Affiliations:** 1 Key Laboratory for Research and Utilization of Characteristic Biological Resources in Southern Yunnan, Honghe University, Mengzi 661199, China; 2 College of Biological and Agricultural Sciences, Honghe University, Mengzi 661199, China

**Keywords:** Honghe Hani Rice Terraces, rice landraces, grain-size traits, genetic diversity, association mapping

## Abstract

The Honghe Hani Rice Terraces System (HHRTS) of Yunnan Province is an important agricultural and cultural heritage landscape. Until now, a large number of local rice landraces have been planted. Mining excellent genes contained in these landraces provides a reference for variety improvement and new variety breeding. In this study, 96 rice landraces collected from the Hani terraces were planted in Honghe Mengzi, Yunnan Province, in 2013, 2014, 2015, and 2021, and five major grain traits were measured and analyzed. The genomic variation of 96 rice landraces was scanned by 201 simple sequence repeat (SSR) markers. The genetic diversity, population structure, and genetic relationships of the natural population were analyzed. The mixed linear model (MLM) method of the TASSEL software was used to analyze the associations between markers and traits. A total of 936 alleles were amplified by 201 pairs of SSR primers. The average number of observed alleles (Na), the effective number of alleles (Ne), Shannon’s information index (I), heterozygosity (H), and the polymorphism information content (PIC) per marker were 4.66, 2.71, 1.08, 0.15, and 0.55, respectively. Ninety-six landraces were divided into two groups by population structure, clustering, and principal component analysis, and *indica* rice was the main group. The coefficients of variation of the five traits ranged from 6.80 to 15.24%, and their broad heritabilities were more than 70%. In addition, there were positive correlations among the same grain traits between different years. Through MLM analysis, 2, 36, 7, 7, and 4 SSR markers were significantly associated with grain length (GL), grain width (GW), grain thickness (GT), grain length–width ratio (LWR), and thousand-grain weight (TGW), respectively. The explanation rates of phenotypic variation were 16.31 (RM449, Chr. 1)—23.51% (RM316, Chr. 9), 10.84 (RM523, Chr. 3; RM161/RM305, Chr. 5)—43.01% (RM5496, Chr. 1), 11.98 (RM161/RM305, Chr. 5)—24.72% (RM275, Chr. 6), 12.68 (RM126, Chr. 8)—36.96% (RM5496, Chr. 1), and 17.65 (RM4499, Chr. 2)—26.32% (RM25, Chr. 8), respectively. The associated markers were distributed on 12 chromosomes of the genome.

## 1. Introduction

Rice (*Oryza sativa* L.), one of the most important food crops in the world, is a staple food for more than half of the world’s population [1]. China is the largest rice producer in the world, with its rice-sown area reaching 30 million hectares (National Bureau of Statistics, 2022). Yunnan Province is recognized as one of the original centers of cultivated rice. It is also the largest center of genetic and ecological diversity of rice germplasm resources in China [2]. The Honghe Hani Rice Terraces System (HHRTS) has a long history as a world heritage cultural landscape and still grows a large number of local rice landraces, especially red rice resources [3]. From ancient times, the Hani people believed that traditional red rice had a higher nutritional value and that consumption of the same made the body healthier. In 2014, Yuanyang terraced red rice was listed as one of the “six famous rices” in Yunnan Province (https://nync.yn.gov.cn/ (accessed on 15 October 2022)). Hani Terrace is located in Southern Yunnan, with its core area being located in Yuanyang County (east longitude 102°27′ to 103°13′, north latitude 22°49′ to 23°19′). The annual average sunshine in this area is 1670 h, with the average temperature being 15.4 °C. Environmental conditions there are suitable for rice cultivation. The Hani people live on a hillside at an altitude of 1400 to 2000 m above sea level. They have a history of rice farming of more than 1500 years with varied rice landraces [4].

Rice grain-size traits usually include grain length (GL), grain width (GW), grain thickness (GT), grain length–width ratio (LWR), and thousand-grain weight (TGW). Rice grain traits are important indicators of both rice quality and yield. Therefore, the study of the inheritance and molecular mechanisms of grain-size traits plays an important role in improving rice yield and quality. Grain size is a typical quantitative trait controlled by polygenes [5]. In recent years, researchers have mapped a large number of quantitative trait loci (QTLs) related to grain type using different genetic populations, including F_2_ populations, backcross populations (BCs), doubling haploid populations (DHs), recombinant inbred lines (RILs), and chromosome segment replacement lines (CSSLs) [6]. Based on incomplete statistics, more than 500 QTLs related to grain size were mapped on 12 chromosomes of rice (http://www.ricedata.cn/index.htm (accessed on 6 March 2022)). Using natural variation among cultivated varieties, researchers cloned several major QTLs related to grain size, including *GW2* [7], *GS2* [8], *TGW2* [9], *OsLG3* [10], *GS3* [11], *qGL3* [12], *qTGW3* [13], *GL4* [14], *GS5* [15], *GW5* [16], *GS6* [17], *GW6* [18], *TGW6* [19], *GL6* [20], *GLW7* [21], *GW7* [22], *GW8* [23], *GS9* [24], and *GW10* [25]. The cloning of these genes updated and improved the grain-size regulatory network, laying a key theoretical foundation and providing genetic resources for high-yield and high-quality rice breeding. Though several major QTLs were identified using a family-based mapping approach, a family population can only identify an allelic variation between two parents, with the optimal allelic variation among species not being found. Association mapping, which utilizes allelic variation in natural populations, can detect many natural allelic variations from germplasm accessions with a much higher resolution compared to linkage mapping. In recent years, excellent allelic variation in grain size in rice was discovered by association analysis [26].

In the present study, a rice association panel containing 96 rice landraces collected from HHRTS in Yunnan was developed to unravel the genetic basis of grain-size traits using 201 simple sequence repeat (SSR) markers. The objectives of the study were to: (i) determine the genetic diversity and population structure of the association panel, (ii) investigate phenotypic traits and their variations in different environments, and (iii) identify significant marker–trait associations for grain-size traits.

## 2. Results

### 2.1. Genetic Diversity Analysis

In total, 936 polymorphic bands were detected from 96 rice landraces by 201 pairs of SSR primers. The observed number of alleles ranged from 2 to 13, with an average of 4.66. The average value of I was 1.08, the value for the primer RM80 being the highest (I = 2.05) and that for the primer RM596 the lowest (I = 0.31). Values of H ranged from 0.00 (RM5994) to 1.00 (RM161, RM1385, RM598, RM479, RM484, RM147, RM319, RM512, RM317, and RM269), with an average of 0.15 (Table 1 and Table S1). As an important index for species genetic diversity evaluation, the PIC values of 201 primer pairs ranged from 0.20 (RM596) to 0.86 (RM206), with an average of 0.55, indicating that these rice landraces from HHRTS had high genetic diversity (Table 1 and Table S1).

### 2.2. Population Structure and Genetic Relationships

**Figure 1:**
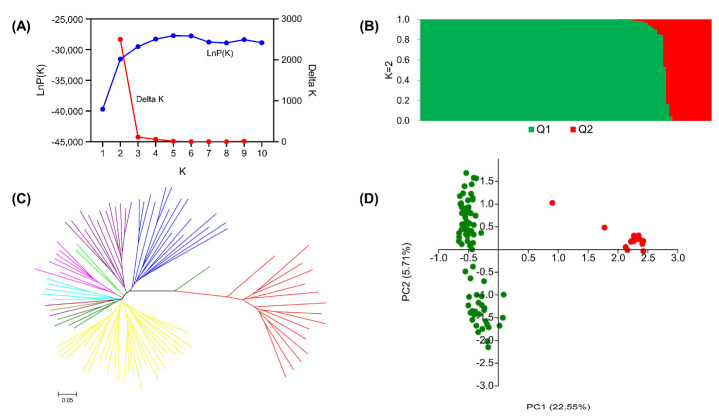
Population structure analysis using Bayesian clustering (**A**,**B**), neighbor joining ((**C**), the red branches represent *japonica* accessions, and the other color branches represent *indica* accessions), and the principal component (**D**) method.

When the population structure of 96 rice landraces in the Hani terraces was analyzed using Structure v.2.3.4, a peak appeared along with an obvious inflection point when K = 2 (Figure 1A). Hence, the 96 rice materials were divided into two groups (Figure 1B), namely, an *indica* rice group (Q1) and a *japonica* rice group (Q2); Q1 contained 81 materials and Q2 contained 15 materials, suggesting that *indica* rice was the main variety in the Hani terraces. To further verify the results of the population structure analysis, we performed clustering and PCA and also divided the rice landraces into two categories (Figure 1C,D). The results were consistent with the population structure analysis based on the STRUCTURE model, indicating that the population structure of this association mapping population was relatively simple and could effectively reduce the influence of population structure on association analysis.

### 2.3. Phenotypic Distribution of Grain-Size Traits

**Figure 2:**
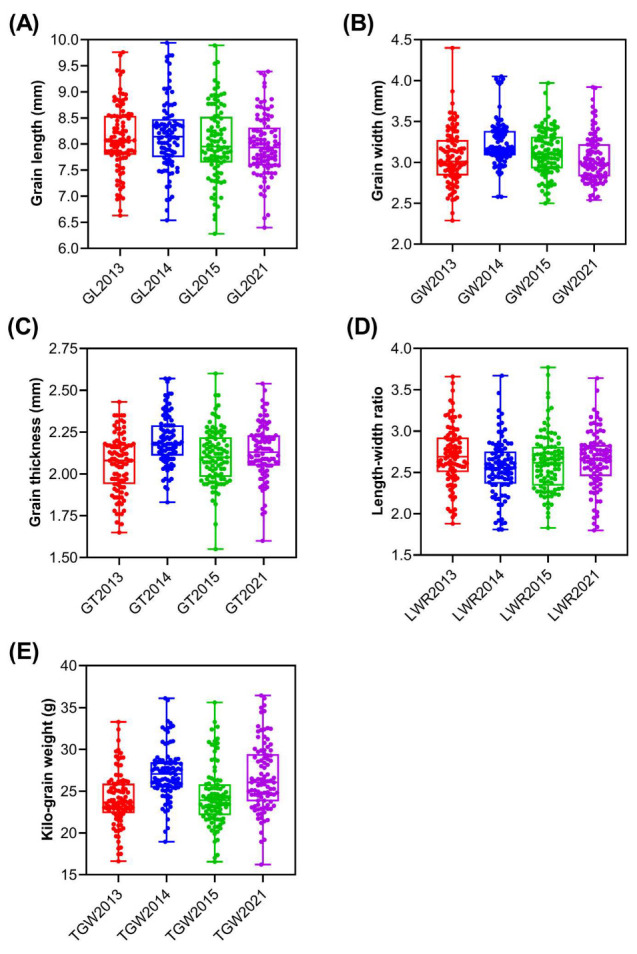
Boxplots (**A**–**E**) of five grain traits in 2013, 2014, 2015, and 2021.

Five grain traits were evaluated in 96 rice landraces in 2013, 2014, 2015, and 2021 (Table 2). The coefficients of variation (CVs) of the five traits ranged from 6.80 to 15.24%. Among the five traits, TGW showed maximum variation, ranging from 11.42 to 15.24%, with abundant variation among different materials. The minimum CV was found in grain thickness, ranging from 6.80 to 7.73%, where the range of variation was relatively narrow. The absolute values for skewness and kurtosis of the five traits in the four environments were less than 1 and 2 (Table 2), respectively, and their distributions were continuous (Figure 2A–E), belonging to typical quantitative traits. The broad-sense heritability ($H_{B}^{2}$) of GL, GW, GT, LWR, and TGW reached 92.63, 87.85, 92.64, 72.39, and 86.11%, respectively, indicating that the grain-size traits were mainly controlled by genetic effects and less by environmental factors.

**Figure 3:**
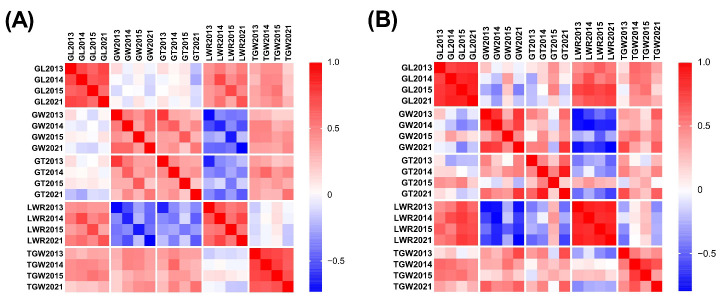
Correlation heatmaps for the *indica* group (**A**) and the *japonica* group (**B**) for five grain traits in 2013, 2014, 2015, and 2021.

The phenotypic data for each trait showed positive correlations in both the *indica* and *japonica* groups between different years (Figure 3 and Table S2). For the *indica* group, the correlation coefficients of GL, GW, GT, LWR, and TGW were 0.57–0.73 (*p* < 0.001), 0.36–0.60 (*p* < 0.001), 0.23–0.46 (*p* < 0.05), 0.52–0.73 (*p* < 0.001), and 0.63–0.76 (*p* < 0.001), respectively. For the *japonica* group, the correlation coefficients of GL, GW, GT, LWR, and TGW were 0.74–0.87 (*p* < 0.01), 0.31–0.80, 0.18–0.61, 0.81–0.88 (*p* < 0.001), and 0.33–0.78, respectively. This indicated relatively high genetic stability in these traits across the years (Table S2). In addition, there were significant correlations among most of the grain-size traits, especially in the *indica* group (Table S2), indicating that these grain traits were interrelated. The largest correlation coefficient among the five traits was −0.80 (LWR and GW, 2021) in the *japonica* group and the smallest was 0.01 (TGW and LWR, 2014) in the *indica* group (Table S2).

### 2.4. Marker–Trait Associations for Grain-Size Traits

**Figure 4:**
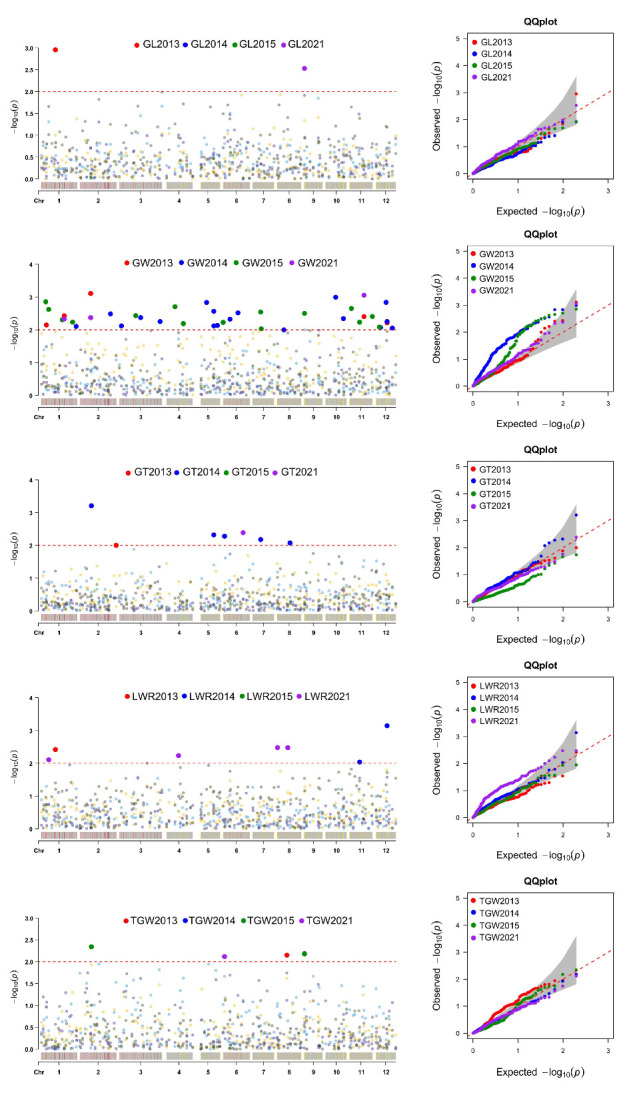
Manhattan plots and quantile–quantile (QQ) plots for grain-size traits based on the MLM model. For the Manhattan plot, the x-axis presents the 12 rice chromosomes and the y-axis the LOD (−log(*p*-value)) values. For the QQ plot, the x-axis presents LOD (−log(*p*-value)) values and the y-axis the expected LOD (−log(*p*-value)) values.

**Figure 5:**
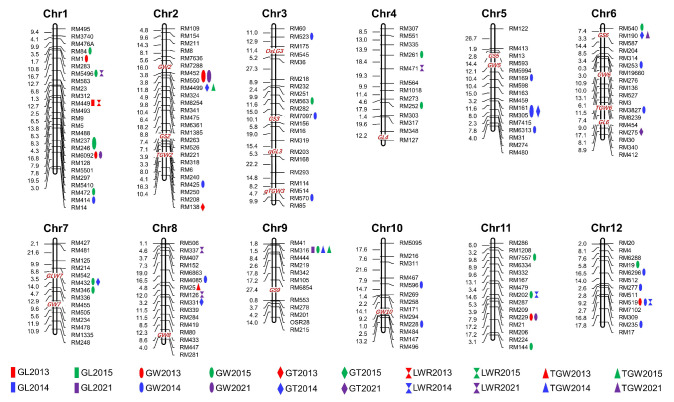
Distribution of significant marker associations for five grain traits. Red, green, blue, and pink shapes indicate 2013, 2014, 2015, and 2021, respectively.

Using the MLM model in Tassel v.3.0, marker–trait association analysis of five grain traits was carried out (Table 3 and Figure 4). In the 2013 and 2021 environments, RM449 (Chr. 1) and RM316 (Chr. 9) were found to be significantly associated with GL traits, explaining 16.31% and 23.51% of the phenotypic variations, respectively (Table 3). For GW, 5, 17, 15, and 3 SSR markers were found to be significantly associated in 2013, 2014, 2015, and 2021, respectively. These loci could explain 10.84 (RM425)—43.01% (RM5496) of the phenotypic variations (Table 3). Of these associated markers, RM6092 (Chr. 1), RM452/RM550 (Chr. 2), RM229 (Chr. 11), and RM519 (Chr. 12) were repeatedly identified in different environments (Figure 5). For GT, 1, 5, and 1 marker-GT association pairs were detected in 2013, 2014, and 2021 respectively, with the marker *R*^2^ ranging from 11.98 (RM161/RM305)—24.72% (RM275) (Table 3 and Figure 5). Seven markers distributed on chromosomes 1, 4, 8, 11, and 12 were found to be associated with LWR, with their *R*^2^ values ranging from 12.68 (RM126) to 36.96% (RM5496) (Table 3 and Figure 5). RM25 (2013), RM316 (2014), RM4499 and RM316 (2015), and RM1901 (2021) were significantly associated with TGW, with their *R*^2^ values ranging from 17.65 to 26.32% (Table 3). It is worth noting that some markers were also associated with more than one phenotype. For instance, RM5496 (Chr. 1), RM202 (Chr. 11), and RM519 (Chr. 12) were found to be associated with GW and LWR, RM449 (Chr. 1) with GL and LWR, RM4499 (Chr. 2) and RM190 (Chr. 6) with GT and TGW, RM161/RM305 (Chr. 5) and RM432 (Chr. 7) with GW and GT, and RM316 (Chr. 9) was found to be significantly associated with GL, GW, and TGW (Figure 5).

## 3. Discussion

### 3.1. Molecular Genetic Diversity

The molecular marker technique can effectively evaluate the genetic diversity and genetic relationships among rice varieties. This is important for the effective protection and utilization of rice germplasm resources [27]. Rice in Yuanyang Hani terraces has been planted for thousands of years. These landraces continued in the long-term cultivation process without being eliminated, indicating that these varieties had strong adaptability and rich genetic variation [28]. However, in recent years, the large-scale popularization of modern bred varieties led to a gradual decrease in the planting area for traditional rice landraces. The analysis of the genetic diversity of rice landraces in the Hani terraces can provide a theoretical basis for the protection and utilization of these rice resources. In this study, a total of 936 polymorphic bands were amplified by 201 SSR markers, with an average of 4.66 bands per primer pair, which was higher compared to a report by Liu et al. (Na = 2.161) [29] but slightly lower compared to a report of Xu et al. (Na = 5.065~5.313) [30]. The high band amplification number showed that rice from the Hani terraces had rich genetic diversity. The average PIC value for SSR markers in this study was similar to that reported in a previous study on 48 pairs of SSR markers (average PIC = 0.652~0.660) [30], which was significantly higher compared to the results of Liu et al. (0.256) [29]. This indicated that the SSR markers used in this study effectively reflected the genetic diversity of rice landraces in the Hani terraces. The I value of 201 SSR markers was higher than 1.000, which further confirmed the rich genetic diversity of the rice landraces in the Hani terraces. Lower heterozygosity (H = 0.15) was consistent with the characteristics of self-pollination and high homozygosity of rice genotypes.

### 3.2. Population Structure and Genetic Relationships

Population structure is an important factor that affects the results of association analysis. Mixing of subpopulations enhances the linkage disequilibrium (LD) intensity of the whole population, resulting in pseudo-association. Hence, the analysis of population genetic structure is the premise of association mapping. In this study, 96 rice landraces in the Hani terraces were analyzed by three different methods: hierarchical Bayesian analysis, NJ cluster analysis, and principal component analysis. Ninety-six landraces were divided into two sub-populations, namely, Q1 and Q2. Q1 was the main group and included 81 landraces (84.38%), while Q2 comprised 15 landraces (15.62%). This was consistent with the results of previous studies on rice landraces in the Hani terraces [3,28,29]. The uncomplicated population structure was suitable for association analysis.

### 3.3. Phenotypic Evaluation

In this study, the heritability of each trait was more than 70%. Higher heritability indicated that the inheritance of grain-size traits was more stable and less affected by environment, which was consistent with the results of Edzesi et al. [31], Feng et al. [32], and Dang et al. [33]. The correlation analysis among characters showed that there was a positive correlation between TGW and GL, GW, and GT in the *indica* group as the main cultivation type of the Hani terraces. However, there was no significant correlation between LWR and TGW in the *indica* or *japonica* group, which was consistent with previous research results [33,34,35,36]. An increase in GL, GW, and GT contributed to an increase in grain weight, with little contribution from LWR. In addition, there was a significant negative correlation between LWR and GT (r = 0.39–0.64, *p* < 0.001) in the *indica* group, which indicated that the long-grain landraces were less thick compared to the round-grain landraces in the Hani terraces.

### 3.4. Marker–Trait Associations

Rice grain size is a typical quantitative trait controlled by multiple genes, which are important indicators of rice yield and appearance. Bi-parental linkage mapping proved effective in mining rice grain-size QTLs. However, the limited number of parents limits the opportunities to recombine the offspring. QTLs cannot be detected when there is no difference in alleles between the parents. Compared to linkage mapping, association analysis uses natural populations as research materials. With natural populations crossing naturally for many generations, recombination is sufficient, the mapping accuracy is higher, and multiple alleles at the same locus can be detected at the same time. In this study, using SSR-trait association analysis, 2, 36, 7, 7, and 4 QTLs controlling GL, GW, GT, LWR, and TGW were detected on 12 rice chromosomes (Figure 5). Compared to previous studies, a few QTLs were similar to known QTLs or genes. RM449 (GL, LWR), RM414 (GW), RM169 (GW), and RM161/RM305 (GW, GT) within the marker intervals RM449-RM237 (*qGL1-2*), RM431-PSM370 (*qGW1-3*), and RM413-RM161 (*qGW5-1*) were detected by Lin et al. [37] using single segment substitution lines (GLU-SSSLs). RM169 on chromosome 5 was found close to the cloned GW5 gene [16], and multiple grain-size QTLs were identified near RM169 [1,26,38,39,40], indicating that this locus played a role in the regulation of grain size in many rice varieties. Zhang et al. [41] used 274 SSR markers to analyze 12 agronomic traits, including GL, GW, LWR, and TGW. The detected loci RM81A (GL), RM144 (GL), RM277 (GL, LWR), RM237 (GW, LWR), RM19 (GW), and RM252 (TGW) were consistent with GL (RM449) and GW (RM144, RM277, RM237, RM19, and RM252) QTLs detected in this study. Zhao et al. [42] identified 53 QTLs related to grain size in two years. The marker intervals of *qKGW1.1*, *qGW2.5*, *qLWR3.2*, *qKGW3.1*, *qGL3.4*, *qLWR3.3*, *qGL6.2*, *qGW6.3*, *qGT9*, *qKGW10*, *qGL11.1*, *qLWR11*, and *qGL11.2* were similar or overlapped with the markers RM6092 (GW, Chr. 1), RM425 (GW, Chr. 2), RM563 (GW, Chr. 3), RM7097 (GW, Chr. 3), RM570 (GW, Chr. 3), RM3827 (GW, Chr. 6), RM316 (GL, GW, TGW, Chr. 6), RM228 (GW, Chr. 10), RM202 (GW, LWR, Chr. 11), and RM229 (GW, Chr. 12) in the present study. Of these, RM452 (GW, Chr. 2), RM425 (GW, Chr. 2), RM570 (GW, Chr. 3), RM169 (GW, Chr. 5), RM190 (GT and TGW, Chr. 6), RM275 (GT, Chr. 6), RM432 (GW and GT, Chr. 7), and RM228 (GW, Chr. 10) were close to the cloned *GW2* [7], *TGW2* [9], *qTGW3* [13], *GW5* [16], *GS6* [17], *TGW6* [19], *GLW7* [21], and *GW10* [25] (Figure 5), respectively. Recently, Zhu et al. [43] finely mapped a TGW QTL, qTGW10-20.8, in the RM228-RM18A region of chromosome 10. However, further verification was required to determine whether the locus detected in this study was related to the cloned gene/QTL allele.

Previous studies revealed that grain-size traits were controlled by a set of QTLs, some of which were QTL clusters. Several SSRs identified in this study overlapped with previous QTL clusters, including RM563 (Chr. 3) and RM169 (Chr. 5), located in QTL clusters RM517-RM411 (Chr. 3) and RM413-RM598 (Chr. 5), studied by Lu et al. [1]. RM7097 (GW) on chromosome 3 was located in the RM411-RM7097 interval controlling seven grain traits, as revealed by Yin et al. [44]. Shi et al. [45] detected *qGL2*, *qGW2*, *qLWR2*, *qGT2*, and *qTGW2a* in the RM322-YP9506 region of chromosome 2, which was consistent with the position of RM452-RM4499, controlling GW, GT, and TGW, detected in this study. Zhang et al. [46] identified a QTL cluster controlling GL, GW, and TGW at the end of the long arm of chromosome 2, which was consistent with the RM138 (GW) locus detected in this study. We detected the RM190 locus controlling GT and TGW on chromosome 6, which was previously identified as being linked to grain size in many populations [47,48,49], including the cloned *GS6* gene, a member of the GRAS gene family, which plays a negative role in regulating the grain size of rice [17]. RM190 is closely linked to the *Wx* gene that controls amylose synthesis, and a few studies showed that rice grain shape was closely related to amylose content [50]. RM5496 (GW, LWR), detected on chromosome 1, explained 43.01% (GW) and 36.96% (LWR) of phenotypic variations. With the *p*-value set to 0.05, this locus was significantly associated with GL in three environments, with *R*^2^ ranging from 31.12 to 32.62%. This indicated that this locus made a major contribution to grain size. RM316, controlling GL (2021), GW (2015), and TGW (2014, 2015), was detected in the short arm of chromosome 9, with *R*^2^ values being above 20%. When *p* < 0.05, RM316 was significantly associated with GL (2014, 2015) and GW (2021). This showed that the RM316 locus played an important role in regulating grain-size traits of rice landraces in the Hani terraces. As no genes controlling grain traits were cloned at the RM316 locus, it is of great significance to further mine the potential genes at this locus.

## 4. Materials and Methods

### 4.1. Rice Material and Phenotyping

Ninety-six rice landraces were collected from Yuanyang County, Honghe Hani, and Yi Autonomous Prefecture of Yunnan Province. Detailed sample information is shown in Table S3. All experimental materials were planted during the rice-growing seasons of 2013, 2014, 2015, and 2021 in the experimental farm of Honghe University, Mengzi, China. A random block design was adopted, and each rice landrace was planted in two rows. Ten plants were planted in each row, with a row spacing of 15 cm × 20 cm between plants and two repetitions. Protective rows were set up around the test field. Field management, including soil fertility and irrigation in the experimental field, was the same in different years. Random samples were harvested, and traits were inspected after natural air-drying. GL, GW, and GT were measured using electronic digital calipers (Guilin Measuring and Cutting Tool Co., Ltd., Guilin, China). Ten grains were measured for each variety, and measurements were repeated five times. Five hundred fully filled grains were randomly selected to measure grain weight, which was repeated twice. With a difference of not more than 5%, grain weight was then converted into TGW.

### 4.2. DNA Extraction and Genotyping

Fresh young leaves of rice were collected at the tillering stage for genomic DNA extraction. DNA was extracted using the cetyltrimethylamine bromide (CTAB) method. DNA concentration was detected by spectrophotometry, and its quality was detected by 1% agarose gel electrophoresis. Finally, DNA was diluted in a working solution of 20 ng/μL. A total of 201 pairs of SSR primers uniformly distributed on rice chromosomes were obtained from the Gramene database (www.gramene.org (accessed on 25 March 2013)). The primers were synthesized by Sangon Biotech Co., Ltd., Shangai. The chromosomes of SSR primers are shown in Table S4. The 10 μL PCR reaction volume contained 10 ng template DNA, 0.2 μM primers, 2.5 mM dNTP, 1.2 μL 10 × PCR buffer, 25 mM MgCl_2_, and 0.5 U rTaq DNA polymerase. The PCR amplification procedure was pre-denatured at 95 °C for 5 min, followed by 30 cycles at 95 °C for 30 s, 55~58 °C for 30 s, 72 °C for 30 s, and extended at 72 °C for 8 min at the end of the cycle. The amplified products were separated by electrophoresis with 8% non-denaturing polyacrylamide gel and stained with 1% silver nitrate [51].

### 4.3. Phenotypic Data Analysis

Excel 2016 was used for data processing and organization. Past v.3.0 software (https://www.nhm.uio.no/english/research/resources/past/ (accessed on 22 November 2022)) was used for phenotypic data descriptive statistics and trait correlation analysis. GraphPad Prism v.8.0 software (GraphPad Prism Software Inc., San Diego, CA, USA) was used to draw a phenotypic data box diagram and correlation heatmap. The following formula was used to estimate the generalized heritability of five traits: ${H_{B}^{2}=\sigma }_{g}^{2}/{(\sigma }_{g}^{2}+\sigma_{e}^{2}/n)$, where $\sigma_{g}^{2}$ is the genetic variance, $\sigma_{e}^{2}$ is the error variance, and *n* is the number of replications [52].

### 4.4. Genetic Diversity, Phylogenetic Analysis, and Population Structure

Polymorphic bands of SSR electrophoresis were recorded and organized based on a previous study [53]. The observed number of alleles (Na), the effective number of alleles (Ne), and Shannon’s information index (I) for each pair of primers were calculated using POPGENE v.1.32 [54]. The polymorphism information content (PIC) and the heterozygosity (H) of each pair of primers were calculated by PowerMarker v.3.25 [55]. The structure of the total population of varieties was analyzed by STRUCTURE v.2.3.4 [56]. The results of the operation repeated five times under different K values were uploaded to Structure Harvester v.0.6.94 (https://taylor0.biology.ucla.edu/structureHarvester/ (accessed on 8 December 2022)) [57] and the optimal number of groups was determined by LnP (K) and Delta (K). Based on the allelic genotype frequency of each locus, the Nei genetic distance, calculated by PowerMarker v.3.25, was used to construct the neighbor-joining (NJ) cluster tree, which was observed using MEGA v.5.0 (http://www.megasoftware.net (accessed on 10 December 2022)). Principle component analysis (PCA) was undertaken using PAST v.3.0.

### 4.5. Association Mapping

The mixed linear model (MLM) in TASSEL v.3.0 was used to analyze the correlation between SSR markers and GL, GW, GT, LWR, and TGW, considering the factors of population structure (Q) and kinship (K) of the materials. The Q value for population structure was calculated using Structure v.2.3.4 and the K value among individuals was calculated using TASSEL v.3.0. It was considered that there was a significant association of the target trait with the marker at a level of *p* < 0.01. The results of the association analysis were visualized by CMplot (https://cran.r-project.org/web/packages/CMplot/ (accessed on 15 December 2022)).

## 5. Conclusions

The genetic diversity, genetic relationships, and population structure of 96 rice landraces in the Hani terraces were analyzed using 201 SSR markers widely distributed on 12 chromosomes. The results showed that rice landraces in the Hani terraces harbored rich genetic diversity. All landraces were divided into two groups, with *indica* rice being the main group. Further, the MLM model of the TASSEL software was used for the SSR marker–trait association analysis of GL, GW, GT, LWR, and TGW in four environments. The results showed that 2, 36, 7, 7, and 4 SSR markers were significantly associated with GL, GW, GT, LWR, and TGW, respectively, with individual phenotypic variations ranging from 10.84 to 43.01%. RM6092 (Chr. 1), RM452/RM550 (Chr. 2), RM316 (Chr. 9), RM229 (Chr. 11), and RM519 (Chr. 12) were detected repeatedly in different environments. Nine SSR markers (RM5496, RM449, RM4499, RM161/RM305, RM190, RM432, RM316, RM202, and RM519) were significantly associated with different grain-size traits. These mapping results provide a theoretical foundation for further fine mapping, cloning, and molecular-assisted breeding of related genes.

## Figures and Tables

**Table 1:** Genetic diversity parameters of rice landraces in the Honghe Hani terraces based on SSR markers.

Parameter	Mean ± SE	Minimum	Maximum
Na	4.66 ± 0.15	2.00	13.00
Ne	2.71 ± 0.09	1.19	6.73
I	1.08 ± 0.03	0.31	2.05
H	0.15 ± 0.02	0.00	1.00
PIC	0.55 ± 0.01	0.20	0.86

Na: observed number of alleles; Ne: effective number of alleles; I: Shannon’s information index; H: heterozygosity; PIC: polymorphism information content.

**Table 2:** Descriptive statistics and heritability in a broad sense for grain-size traits in rice.

Traits	Year	Mean ± SE	Minimum	Maximum	SD	Skewness	Kurtosis	CV (%)	$H_{B}^{2}$ (%)
GL (mm)	2013	8.12 ± 0.06	6.63	9.76	0.63	0.11	0.13	7.80	92.63
	2014	8.17 ± 0.07	6.54	9.94	0.67	0.34	0.42	8.20	
	2015	8.03 ± 0.07	6.28	9.89	0.70	0.10	−0.04	8.74	
	2021	7.97 ± 0.06	6.40	9.39	0.59	0.14	0.31	7.39	
GW (mm)	2013	3.06 ± 0.03	2.29	4.40	0.33	0.69	1.84	10.91	87.85
	2014	3.24 ± 0.03	2.58	4.05	0.28	0.95	1.94	8.51	
	2015	3.11 ± 0.03	2.50	3.97	0.28	0.25	0.32	8.89	
	2021	3.04 ± 0.03	2.54	3.92	0.30	0.85	0.58	9.72	
GT (mm)	2013	2.06 ± 0.02	1.65	2.43	0.17	−0.23	−0.49	8.21	92.64
	2014	2.20 ± 0.02	1.83	2.57	0.15	0.30	0.07	6.80	
	2015	2.11 ± 0.02	1.55	2.60	0.17	−0.01	1.11	7.88	
	2021	2.13 ± 0.02	1.60	2.54	0.17	−0.28	0.72	7.73	
LWR	2013	2.71 ± 0.04	1.88	3.66	0.36	0.08	0.12	13.38	72.39
	2014	2.56 ± 0.04	1.81	3.67	0.35	0.22	0.85	13.51	
	2015	2.62 ± 0.04	1.83	3.77	0.36	0.63	0.93	13.65	
	2021	2.65 ± 0.03	1.80	3.64	0.34	−0.14	0.63	12.90	
TGW (g)	2013	24.06 ± 0.33	16.62	33.28	3.19	0.33	0.53	13.25	86.11
	2014	27.16 ± 0.32	18.96	36.11	3.10	0.38	0.93	11.42	
	2015	24.42 ± 0.37	16.56	35.61	3.60	0.72	0.76	14.75	
	2021	26.64 ± 0.41	16.22	36.45	4.06	0.34	−0.11	15.24	

**Table 3:** SSR markers significantly associated with five grain traits in four years.

Traits	Year	Locus	Chr.	*p*-Value	*R*^2^ (%)
GL	2013	RM449	1	1.11 × 10^−3^	16.31
	2021	RM316	9	2.95 × 10^−3^	23.51
GW	2013	RM6092	1	3.74 × 10^−3^	26.79
	2013	RM1	1	7.10 × 10^−3^	37.26
	2013	RM452/RM550	2	7.77 × 10^−4^	19.04
	2013	RM229	11	3.96 × 10^−3^	25.03
	2013	RM519	12	6.11 × 10^−3^	26.57
	2014	RM414	1	7.85 × 10^−3^	29.24
	2014	RM425	2	3.26 × 10^−3^	12.86
	2014	RM523	3	7.57 × 10^−3^	10.84
	2014	RM7097	3	4.24 × 10^−3^	25.92
	2014	RM570	3	5.60 × 10^−3^	28.92
	2014	RM169	5	1.46 × 10^−3^	28.83
	2014	RM161/RM305	5	2.72 × 10^−3^	13.31
	2014	RM6313	5	7.32 × 10^−3^	10.92
	2014	RM253	6	4.74 × 10^−3^	28.10
	2014	RM3827	6	3.04 × 10^−3^	18.19
	2014	RM4085	8	9.98 × 10^−3^	19.55
	2014	RM596	10	1.01 × 10^−3^	16.31
	2014	RM228	10	4.54 × 10^−3^	36.56
	2014	RM6296	12	8.51 × 10^−3^	11.21
	2014	RM277	12	1.45 × 10^−3^	15.88
	2014	RM519	12	5.59 × 10^−3^	25.90
	2014	RM235	12	8.83 × 10^−3^	42.32
	2015	RM84	1	1.39 × 10^−3^	40.03
	2015	RM5496	1	2.38 × 10^−3^	43.01
	2015	RM246/RM237	1	4.95 × 10^−3^	25.54
	2015	RM472	1	5.82 × 10^−3^	21.38
	2015	RM563	3	3.70 × 10^−3^	18.1
	2015	RM261	4	1.97 × 10^−3^	19.57
	2015	RM252	4	6.47 × 10^−3^	26.12
	2015	RM540	6	5.90 × 10^−3^	27.18
	2015	RM432	7	2.86 × 10^−3^	21.85
	2015	RM346	7	9.28 × 10^−3^	23.7
	2015	RM316	9	3.15 × 10^−3^	22.8
	2015	RM7557	11	2.22 × 10^−3^	19.43
	2015	RM202	11	5.85 × 10^−3^	18.81
	2015	RM144	11	3.92 × 10^−3^	30.78
	2015	RM19	12	8.22 × 10^−3^	20.66
	2021	RM6092	1	4.67 × 10^−3^	27.28
	2021	RM452/RM550	2	4.26 × 10^−3^	15.57
	2021	RM229	11	8.79 × 10^−4^	29.01
GT	2013	RM138	2	9.95 × 10^−3^	12.73
	2014	RM4499	2	6.19 × 10^−4^	23.47
	2014	RM161/RM305	5	4.79 × 10^−3^	11.98
	2014	RM190	6	5.26 × 10^−3^	18.81
	2014	RM432	7	6.65 × 10^−3^	19.21
	2014	RM331	8	8.42 × 10^−3^	23.81
	2021	RM275	6	4.10 × 10^−3^	24.72
LWR	2013	RM449	1	3.81 × 10^−3^	13.18
	2014	RM202	11	9.19 × 10^−3^	19.02
	2014	RM519	12	7.16 × 10^−4^	32.94
	2021	RM5496	1	7.80 × 10^−3^	36.96
	2021	RM471	4	5.82 × 10^−3^	24.54
	2021	RM337	8	3.32 × 10^−3^	20.04
	2021	RM126	8	3.33 × 10^−3^	12.68
TGW	2013	RM25	8	7.09 × 10^−3^	26.32
	2014	RM316	9	6.57 × 10^−3^	20.52
	2015	RM4499	2	4.56 × 10^−3^	17.65
	2015	RM316	9	6.63 × 10^−3^	20.72
	2021	RM190	6	7.62 × 10^−3^	17.88

## Data Availability

The datasets supporting the conclusions of this article are included within the article.

## Supplementary Materials

The following supporting information can be downloaded at: https://www.mdpi.com/article/10.3390/plants12081678/s1, Table S1: Genetic diversity parameters of 96 rice landraces collected from the Hani terraces based on 201 SSR markers; Table S2: Correlation analysis of five grain-size traits of Hani terrace rice landraces in four environments; Table S3: Codes, names, and collection places of 96 rice landraces from the Honghe Hani Rice Terraces System; Table S4: Chromosome information for 201 pairs of SSR markers.
