# Supplementary material for: Genetic Diversity and Association Mapping of Grain-Size Traits in Rice Landraces from the Honghe Hani Rice Terraces System in Yunnan Province
Source: Plants (Basel). 2023 Apr 17;12(8):1678. doi: 10.3390/plants12081678 (PMC10146266; doi:10.3390/plants12081678)
Supplement: Supplementary file 1 [file plants-12-01678-s001.zip › Table S1.pdf]

Table S1. Genetic diversity parameters of 96 rice landraces collected from the Hani terraces based on 201 SSR markers.

Locus	Na	Ne	I	H	PIC	Locus	Na	Ne	I	H	PIC
RM495	3	1.456	0.514	0.010	0.267	RM190	3	2.442	0.965	0.125	0.505
RM3740	3	1.910	0.766	0.063	0.458	RM587	4	1.501	0.650	0.063	0.335
RM476A	4	2.435	1.087	0.385	0.650	RM204	12	3.543	1.744	0.083	0.731
RM84	6	3.640	1.439	0.094	0.738	RM314	4	1.806	0.717	0.010	0.364
RM1	7	5.352	1.792	0.083	0.805	RM253	5	2.363	1.091	0.073	0.557
RM283	3	1.975	0.711	0.031	0.377	RM19660	9	6.086	1.926	0.146	0.834
RM5496	8	5.986	1.879	0.083	0.819	RM276	4	2.764	1.141	0.042	0.679
RM583	6	4.342	1.587	0.063	0.739	RM136	3	1.795	0.786	0.063	0.400
RM23	8	4.901	1.738	0.135	0.772	RM527	5	2.514	1.176	0.063	0.596
RM312	6	3.243	1.380	0.760	0.727	RM3	7	3.080	1.317	0.042	0.700
RM449	2	1.491	0.511	0.010	0.372	RM3827	3	1.333	0.492	0.031	0.233
RM493	6	3.662	1.490	0.063	0.747	RM8239	3	1.494	0.565	0.021	0.412
RM9	4	2.685	1.170	0.021	0.590	RM454	3	1.415	0.548	0.031	0.448
RM5	5	3.426	1.375	0.052	0.701	RM275	7	2.658	1.178	0.052	0.577
RM488	5	3.103	1.268	0.031	0.660	RM30	3	2.016	0.872	0.677	0.451
RM237	4	2.535	1.038	0.031	0.656	RM340	5	1.507	0.663	0.042	0.385
RM246	5	3.135	1.263	0.125	0.633	RM412	4	2.213	0.975	0.083	0.503
RM6092	4	2.520	1.063	0.031	0.655	RM427	4	2.179	0.959	0.010	0.498
RM128	2	1.385	0.451	0.031	0.286	RM481	12	3.729	1.806	0.865	0.752
RM5501	6	3.212	1.354	0.260	0.711	RM125	6	1.700	0.836	0.021	0.383
RM297	11	3.302	1.684	0.823	0.712	RM214	4	2.629	1.114	0.115	0.573
RM5410	4	1.618	0.716	0.052	0.390	RM542	6	2.752	1.239	0.115	0.589
RM472	4	3.475	1.312	0.083	0.683	RM432	3	2.166	0.861	0.104	0.494

RM414	5	2.782	1.168	0.063	0.660	RM346	5	2.861	1.146	0.094	0.618
RM14	7	5.893	1.845	0.448	0.837	RM336	7	3.972	1.523	0.156	0.706
RM109	6	1.909	0.955	0.010	0.454	RM455	2	1.510	0.521	0.021	0.326
RM154	5	1.881	0.920	0.042	0.446	RM505	3	2.292	0.901	0.802	0.583
RM211	2	1.270	0.369	0.031	0.207	RM234	6	3.013	1.290	0.104	0.678
RM8	4	2.382	1.033	0.208	0.511	RM478	3	2.880	1.077	0.083	0.588
RM7636	4	2.648	1.090	0.042	0.569	RM1335	5	3.279	1.340	0.052	0.711
RM7288	13	4.244	1.858	0.177	0.768	RM248	8	5.536	1.826	0.052	0.828
RM452	2	1.957	0.682	0.010	0.533	RM506	5	3.360	1.351	0.052	0.673
RM550	3	2.435	0.959	0.031	0.535	RM337	4	2.155	0.994	0.125	0.486
RM4499	4	1.219	0.405	0.010	0.222	RM407	4	3.494	1.309	0.083	0.661
RM324	3	2.546	1.003	0.115	0.562	RM152	5	2.416	1.036	0.885	0.588
RM8254	8	4.426	1.681	0.865	0.765	RM6863	3	2.418	0.983	0.083	0.618
RM341	6	3.551	1.509	0.135	0.687	RM4085	4	2.046	0.975	0.104	0.515
RM475	4	1.734	0.829	0.042	0.407	RM25	7	2.576	1.299	0.177	0.582
RM6361	4	2.298	0.991	0.042	0.534	RM126	2	1.686	0.597	0.021	0.340
RM1385	5	1.349	0.582	0.000	0.319	RM331	4	2.758	1.137	0.115	0.649
RM263	6	5.017	1.688	0.115	0.786	RM339	9	5.457	1.881	0.135	0.819
RM526	3	1.970	0.857	0.302	0.517	RM284	2	1.427	0.476	0.010	0.341
RM221	2	1.471	0.500	0.083	0.354	RM419	3	1.264	0.413	0.021	0.211
RM318	3	1.733	0.696	0.042	0.371	RM80	11	6.450	2.052	0.188	0.837
RM6	4	2.332	1.047	0.052	0.568	RM433	6	3.365	1.432	0.031	0.659
RM240	7	2.082	1.042	0.073	0.471	RM447	6	4.399	1.599	0.896	0.780
RM425	2	1.362	0.436	0.021	0.247	RM281	6	2.882	1.268	0.021	0.639
RM250	2	1.448	0.488	0.010	0.423	RM41	2	1.692	0.599	0.490	0.485
RM208	3	1.937	0.719	0.063	0.456	RM316	5	1.570	0.779	0.208	0.360

RM138	3	1.615	0.693	0.021	0.360	RM444	9	6.115	1.984	0.094	0.843
RM60	3	1.301	0.426	0.052	0.225	RM219	9	4.847	1.824	0.240	0.796
RM523	2	1.371	0.442	0.010	0.234	RM342	5	3.321	1.309	0.177	0.686
RM175	5	2.599	1.129	0.938	0.539	RM105	8	3.057	1.422	0.031	0.669
RM545	6	3.852	1.522	0.042	0.716	RM6854	3	1.824	0.767	0.021	0.391
RM36	5	2.545	1.212	0.083	0.589	RM553	5	2.917	1.237	0.083	0.636
RM218	5	2.623	1.153	0.104	0.596	RM278	5	3.482	1.377	0.052	0.679
RM232	9	6.218	1.978	0.125	0.828	RM201	4	2.169	1.027	0.052	0.532
RM251	5	4.266	1.515	0.042	0.766	OSR28	9	2.817	1.488	0.104	0.670
RM563	4	2.559	1.092	0.010	0.586	RM215	3	2.899	1.082	0.063	0.626
RM282	5	2.664	1.202	0.010	0.582	RM5095	9	6.677	2.043	0.052	0.854
RM7097	5	2.659	1.253	0.063	0.588	RM216	3	2.485	0.997	0.083	0.577
RM156	4	3.442	1.290	0.823	0.677	RM311	7	4.945	1.699	0.865	0.807
RM16	5	2.129	1.068	0.010	0.537	RM467	4	1.756	0.857	0.010	0.472
RM319	3	1.740	0.762	0.000	0.412	RM596	3	1.188	0.313	0.167	0.198
RM203	2	1.800	0.637	0.563	0.428	RM269	4	2.791	1.097	0.000	0.671
RM168	6	1.725	0.912	0.042	0.401	RM258	5	2.662	1.176	0.042	0.628
RM293	2	1.994	0.692	0.073	0.456	RM171	4	2.649	1.056	0.750	0.544
RM114	3	2.142	0.907	0.646	0.586	RM294	2	1.484	0.508	0.031	0.289
RM514	3	2.071	0.871	0.094	0.550	RM228	6	3.881	1.507	0.115	0.714
RM570	6	3.567	1.453	0.104	0.676	RM484	3	1.693	0.739	0.000	0.386
RM85	3	1.641	0.698	0.031	0.364	RM147	3	1.790	0.756	0.000	0.424
RM307	5	3.629	1.403	0.156	0.750	RM496	4	1.979	0.932	0.021	0.540
RM551	7	3.399	1.461	0.052	0.669	RM286	6	3.765	1.494	0.073	0.712
RM335	10	5.727	1.990	0.698	0.834	RM1208	4	3.256	1.232	0.135	0.692
RM261	3	1.782	0.705	0.052	0.377	RM7557	3	1.690	0.727	0.021	0.393

RM471	5	3.318	1.312	0.073	0.645	RM6334	2	1.248	0.350	0.010	0.212
RM564	5	2.681	1.168	0.010	0.605	RM332	5	2.938	1.223	0.083	0.616
RM1018	3	2.309	0.949	0.010	0.498	RM167	3	1.972	0.858	0.094	0.455
RM273	3	1.395	0.550	0.031	0.264	RM479	2	1.977	0.687	0.000	0.429
RM252	4	1.496	0.689	0.042	0.447	RM202	4	1.629	0.748	0.021	0.381
RM303	7	3.790	1.578	0.031	0.737	RM287	5	3.463	1.359	0.104	0.668
RM317	4	2.094	0.931	0.000	0.470	RM209	3	1.857	0.769	0.010	0.433
RM348	3	1.446	0.523	0.021	0.267	RM229	5	2.152	1.109	0.083	0.529
RM127	2	1.634	0.576	0.021	0.328	RM21	7	3.104	1.501	0.156	0.701
RM122	3	2.346	0.969	0.073	0.647	RM206	9	6.727	2.040	0.094	0.857
RM413	5	3.166	1.305	0.083	0.694	RM224	4	2.771	1.135	0.063	0.635
RM13	5	2.983	1.242	0.125	0.608	RM144	5	3.534	1.427	0.219	0.748
RM593	4	3.786	1.355	0.010	0.686	RM20	6	3.127	1.346	0.708	0.713
RM5994	3	2.359	0.934	1.000	0.486	RM4	5	4.553	1.559	0.750	0.770
RM169	4	3.659	1.339	0.094	0.710	RM6288	3	1.939	0.763	0.042	0.393
RM598	3	1.798	0.675	0.000	0.354	RM19	6	2.980	1.198	0.042	0.654
RM163	5	2.357	1.175	0.073	0.555	RM6296	2	1.394	0.457	0.021	0.354
RM459	3	1.413	0.491	0.010	0.362	RM512	3	2.069	0.776	0.000	0.403
RM161	3	1.297	0.454	0.000	0.245	RM277	2	1.356	0.432	0.021	0.316
RM305	2	1.371	0.442	0.010	0.234	RM511	2	1.484	0.508	0.031	0.289
RM7415	4	2.132	0.834	0.781	0.473	RM519	5	3.643	1.442	0.052	0.712
RM6313	2	1.358	0.433	0.021	0.229	RM7102	7	3.711	1.514	0.563	0.745
RM31	4	2.997	1.204	0.073	0.707	RM309	3	2.566	1.018	0.052	0.631
RM274	2	1.371	0.442	0.010	0.234	RM235	8	4.520	1.698	0.604	0.792
RM480	5	1.935	1.011	0.073	0.482	RM17	4	2.959	1.228	0.135	0.631
RM540	5	2.988	1.288	0.198	0.659	Average	4.657	2.714	1.077	0.145	0.553

Na: observed number of alleles; Ne: effective number of alleles; I: Shannon’s information index; H: heterozygosity; PIC: polymorphism information content
