# Supplementary material for: Genetic Diversity and Association Mapping of Grain-Size Traits in Rice Landraces from the Honghe Hani Rice Terraces System in Yunnan Province
Source: Plants (Basel). 2023 Apr 17;12(8):1678. doi: 10.3390/plants12081678 (PMC10146266; doi:10.3390/plants12081678)
Supplement: Supplementary file 1 [file plants-12-01678-s001.zip › Table S2.pdf]

Table S2. Correlation analysis among 5 grain-size traits of Hani terraces rice landraces under four environments, *indica* group (below diagonal) and *japonica* group (above diagonal).

	GL2013	GL2014	GL2015	GL2021	GW2013	GW2014	GW2015	GW2021	GT2013	GT2014	GT2015	GT2021	LWR2013	LWR2014	LWR2015	LWR2021	TGW2013	TGW2014	TGW2015	TGW2021
GL2013	1.00	0.74**	0.78***	0.79***	0.23	-0.04	0.09	-0.04	0.09	0.27	0.42	0.13	0.44	0.51	0.63*	0.51*	0.13	0.59*	0.54*	0.36
GL2014	0.71***	1.00	0.84***	0.87***	-0.05	-0.19	0.38	-0.08	-0.28	0.25	0.39	0.05	0.52*	0.70**	0.49	0.58*	0.23	0.56*	0.51*	0.28
GL2015	0.60***	0.65***	1.00	0.86***	-0.255	-0.39	0.22	-0.40	-0.21	0.17	0.49	-0.17	0.73**	0.77***	0.72**	0.77***	-0.04	0.46	0.55*	0.15
GL2021	0.73***	0.70***	0.57***	1.00	-0.07	-0.30	0.11	-0.18	-0.20	0.05	0.18	-0.09	0.58*	0.68**	0.69**	0.73**	0.07	0.42	0.42	0.30
GW2013	0.14	-0.02	-0.11	0.03	1.00	0.80***	0.31	0.79***	0.49	0.17	-0.02	0.57*	-0.77***	-0.59*	-0.45	-0.58*	0.38	0.33	0.12	0.63*
GW2014	-0.04	-0.08	0.00	-0.09	0.52***	1.00	0.52*	0.75**	0.19	0.32	-0.12	0.44	-0.75**	-0.79***	-0.69**	-0.69**	0.27	0.32	0.16	0.46
GW2015	0.06	0.03	-0.02	0.11	0.43***	0.36***	1.00	0.46	0.02	0.46	0.17	0.40	-0.26	-0.18	-0.52*	-0.27	0.47	0.55*	0.33	0.49
GW2021	-0.04	-0.12	-0.13	-0.03	0.60***	0.60***	0.38***	1.00	0.49	0.12	-0.22	0.78***	-0.76**	-0.61*	-0.67**	-0.80***	0.72**	0.36	0.18	0.63**
GT2013	0.14	-0.03	0.00	0.11	0.78***	0.53***	0.38***	0.37***	1.00	0.26	0.22	0.61*	-0.42	-0.28	-0.24	-0.49	0.39	0.03	-0.10	0.28
GT2014	0.03	0.02	0.06	-0.03	0.34**	0.63***	0.35**	0.43***	0.46***	1.00	0.44	0.48	0.00	-0.02	-0.17	-0.04	0.03	0.40	0.23	0.44
GT2015	0.10	0.06	0.09	0.04	0.32**	0.32**	0.65***	0.32**	0.36**	0.37***	1.00	0.18	0.28	0.41	0.29	0.25	-0.11	0.22	0.33	-0.02
GT2021	-0.18	-0.25*	-0.10	-0.11	0.24*	0.45***	0.30**	0.53***	0.34**	0.42***	0.23*	1.00	-0.46	-0.27	-0.44	-0.60*	0.70**	0.36	0.23	0.63*
LWR2013	0.43***	0.45***	0.40***	0.40***	-0.72***	-0.52***	-0.27**	-0.43***	-0.64***	-0.34**	-0.19	-0.35**	1.00	0.87***	0.85***	0.88***	-0.29	0.06	0.25	-0.38
LWR2014	0.52***	0.72***	0.47***	0.55***	-0.34**	-0.64***	-0.21	-0.45***	-0.34**	-0.39***	-0.12	-0.46***	0.66***	1.00	0.81***	0.83***	-0.08	0.09	0.23	-0.27
LWR2015	0.38***	0.45***	0.71***	0.34**	-0.40***	-0.34**	-0.67***	-0.41***	-0.29**	-0.25*	-0.39***	-0.29**	0.52***	0.56***	1.00	0.88***	-0.36	0.03	0.26	-0.25
LWR2021	0.50***	0.57***	0.46***	0.68***	-0.38***	-0.51***	-0.20	-0.74***	-0.20	-0.37***	-0.14	-0.48***	0.58***	0.73***	0.53***	1.00	-0.45	0.00	0.13	-0.27
TGW2013	0.32***	0.27*	0.35**	0.34**	0.31**	0.49***	0.47***	0.38***	0.39***	0.46***	0.37***	0.36***	-0.15	-0.10	-0.11	-0.09	1.00	0.52*	0.33	0.45
TGW2014	0.43***	0.47***	0.46***	0.41***	0.28*	0.48***	0.46***	0.37***	0.34**	0.60***	0.32**	0.28*	-0.02	0.01	-0.03	-0.03	0.75***	1.00	0.78***	0.67**
TGW2015	0.44***	0.46***	0.54***	0.48***	0.17	0.30**	0.37***	0.34**	0.23*	0.40***	0.40***	0.29**	0.12	0.14	0.08	0.04	0.72***	0.76***	1.00	0.37
TGW2021	0.17	0.23*	0.21	0.30**	0.17	0.34**	0.44***	0.46***	0.27*	0.48***	0.32***	0.55***	-0.02	-0.04	-0.16	-0.17	0.65***	0.63***	0.68***	1.00

*, ** and *** represent the coefficient is significant at the 0.05, 0.01 and 0.001 levels, respectively.
