# Supplementary material for: Genetic Diversity and Association Mapping of Grain-Size Traits in Rice Landraces from the Honghe Hani Rice Terraces System in Yunnan Province
Source: Plants (Basel). 2023 Apr 17;12(8):1678. doi: 10.3390/plants12081678 (PMC10146266; doi:10.3390/plants12081678)
Supplement: Supplementary file 1 [file plants-12-01678-s001.zip › Table S3.pdf]

Table S3. Code, name and collecting place of 96 rice landraces from Honghe Hani rice terraces system.

Code	Name	Collecting place	Code	Name	Collecting place
1	Dumiaogu	Shangxincheng Township	49	Maichou	Eza Township
2	Anxinggu	Huangmaoling Township	50	Nuogu	Ganiang Township
3	Rongrenche	Niujiaozhai Township	51	Honggu	Ganiang Township
4	Rongrenrenche	Niujiaozhai Township	52	Duominjiahelue	Eza Township
5	Chemaouzheng	Missing source	53	zigu	Missing source
6	Honggunuo	Shangxincheng Township	54	Pozhugu	Missing source
7	Bendidalixiang	Shangxincheng Township	55	Chengke	Missing source
8	Heigunuo	Shangxincheng Township	56	Chefu	Xinjie Town
9	Zimi	Missing source	57	Zimi	Missing source
10	Poluo	Shangxincheng Township	58	Jiagu	Missing source
11	Gaoshanhonggu	Shangxincheng Township	59	Chengni	Missing source
12	Luoge	Niujiaozhai Township	60	Laogenghongmi	Huangcaoling Township
13	Baijiaolaogeng	Xinjie Town	61	Dianmeng2	Shalatuoxiang Township
14	Chemanche	Niujiaozhai Township	62	Shiouqian	Huangcaoling Township
15	Chena	Missing source	63	Chenilongge	Huangcaoling Township
16	Maoche	Niujiaozhai Township	64	Helingu	Eza Township
17	Bianhao3	Xiaoxinjie Township	65	Wupucheni	Eza Township
18	Laopinzhonghongmi	Panzhihua Township	66	Apenpenche	Huangcaoling Township
19	Huagu	Huangmaoling Township	67	Kounihelue	Huangcaoling Township
20	Taiyanggu	Shangxincheng Township	68	Hexi41	Ganiang Township
21	Yigu	Xinjie Town	69	Honggu	Daping Township
22	Longgenuo	Shangxincheng Township	70	Haninuo	Daping Township
23	Chejiaojiaoer	Missing source	71	Zajiaonuogu1	Shalatuoxiang Township
24	Poxinggu	Xiaoxinjie Township	72	Laowunuo	Daping Township
25	Bendihonggu1	Missing source	73	Zajiaonuogu2	Missing source
26	Gutianche	Missing source	74	Liuyuegu	Xinjie Town
27	Lvjiaogu	Xinjie Town	75	Zajiaonuogu3	Shalatuoxiang Township
28	Bendihonggu2	Shangxincheng Township	76	Laomujigu	Shangxincheng Township
29	Luogeihonglue	Xinjie Town	77	Hongnuogu	Shalatuoxiang Township

30	Laopinzhong	Panzhihua Township	78	Zajiaohonggu	Shalatuoxiang Township
31	Pingcunnuogu	Shangxincheng Township	79	Gangu	Daping Township
32	Dalonggu	Eza Township	80	Shuihandao	Huangmaoling Township
33	Longshanlinggu	Xinjie Town	81	laopinzhongbaigu	Shalatuoxiang Township
34	Yaduogu	Xinjie Town	82	Yaorengu	Daping Township
35	Chenu	Shengcun Township	83	Honggu	Daping Township
36	Qixing	Shangxincheng Township	84	Wopuchezuo	Xinjie Town
37	Jjianshuigu	Xinjie Town	85	Zhenzhumigu	Shangxincheng Township
38	Huaaigu	Xinjie Town	86	Baipinuo	Xinjie Town
39	Huagu	Shengcun Township	87	Bianhao6	Xiaoxinjie Township
40	Lengshuigu	Daping Township	88	Baimi	Panzhihua Township
41	Chena	Xinjie Town	89	Zinuo	Missing source
42	Gaoshanhonggu	Missing source	90	Cheran	Xinjie Town
43	Maoche	Xinjie Town	91	Manchehonglue	Xinjie Town
44	Honggunuo	Missing source	92	Chejiagu	Xinjie Town
45	Laogengnuo	Missing source	93	Hongpinuo	Missing source
46	Aizheranche	Xinjie Town	94	Huangpinuo	Xinjie Town
47	Cesuogu	Daping Township	95	Yueliangu	Xinjie Town
48	Kenunuxiang	Eza Township	96	Baigu	Shengcun Township
