# Supplementary material for: Genetic Diversity and Association Mapping of Grain-Size Traits in Rice Landraces from the Honghe Hani Rice Terraces System in Yunnan Province
Source: Plants (Basel). 2023 Apr 17;12(8):1678. doi: 10.3390/plants12081678 (PMC10146266; doi:10.3390/plants12081678)
Supplement: Supplementary file 1 [file plants-12-01678-s001.zip › Table S4.pdf]

Table S4. Chromosome information of 201 pairs of SSR markers.

Primer name	Chromosome	Primer name	Chromosome	Primer name	Chromosome
RM495	1	RM114	3	RM407	8
RM3740	1	RM514	3	RM152	8
RM476A	1	RM570	3	RM6863	8
RM84	1	RM85	3	RM4085	8
RM1	1	RM307	4	RM25	8
RM283	1	RM551	4	RM126	8
RM5496	1	RM335	4	RM331	8
RM583	1	RM261	4	RM339	8
RM23	1	RM471	4	RM284	8
RM312	1	RM564	4	RM419	8
RM449	1	RM1018	4	RM80	8
RM493	1	RM273	4	RM433	8
RM9	1	RM252	4	RM447	8
RM5	1	RM303	4	RM281	8
RM488	1	RM317	4	RM41	9
RM237	1	RM348	4	RM316	9
RM246	1	RM127	4	RM444	9
RM6092	1	RM122	5	RM219	9
RM128	1	RM413	5	RM342	9
RM5501	1	RM13	5	RM105	9
RM297	1	RM593	5	RM6854	9
RM5410	1	RM5994	5	RM553	9
RM472	1	RM169	5	RM278	9
RM414	1	RM598	5	RM201	9
RM14	1	RM163	5	OSR28	9
RM109	2	RM459	5	RM215	9
RM154	2	RM161	5	RM5095	10
RM211	2	RM305	5	RM216	10
RM8	2	RM7415	5	RM311	10
RM7636	2	RM6313	5	RM467	10
RM7288	2	RM31	5	RM596	10
RM452	2	RM274	5	RM269	10
RM550	2	RM480	5	RM258	10
RM4499	2	RM540	6	RM171	10
RM324	2	RM190	6	RM294	10
RM8254	2	RM587	6	RM228	10
RM341	2	RM204	6	RM484	10
RM475	2	RM314	6	RM147	10
RM6361	2	RM253	6	RM496	10
RM1385	2	RM19660	6	RM286	11
RM263	2	RM276	6	RM1208	11

RM526	2	RM136	6	RM7557	11
RM221	2	RM527	6	RM6334	11
RM318	2	RM3	6	RM332	11
RM6	2	RM3827	6	RM167	11
RM240	2	RM8239	6	RM479	11
RM425	2	RM454	6	RM202	11
RM250	2	RM275	6	RM287	11
RM208	2	RM30	6	RM209	11
RM138	2	RM340	6	RM229	11
RM60	3	RM412	6	RM21	11
RM523	3	RM427	7	RM206	11
RM175	3	RM481	7	RM224	11
RM545	3	RM125	7	RM144	11
RM36	3	RM214	7	RM20	12
RM218	3	RM542	7	RM4	12
RM232	3	RM432	7	RM6288	12
RM251	3	RM346	7	RM19	12
RM563	3	RM336	7	RM6296	12
RM282	3	RM455	7	RM512	12
RM7097	3	RM505	7	RM277	12
RM156	3	RM234	7	RM511	12
RM16	3	RM478	7	RM519	12
RM319	3	RM1335	7	RM7102	12
RM203	3	RM248	7	RM309	12
RM168	3	RM506	8	RM235	12
RM293	3	RM337	8	RM17	12
